# Human Gut-Microbiota Interaction in Neurodegenerative Disorders and Current Engineered Tools for Its Modeling

**DOI:** 10.3389/fcimb.2020.00297

**Published:** 2020-07-07

**Authors:** Florencia Andrea Ceppa, Luca Izzo, Lorenzo Sardelli, Ilaria Raimondi, Marta Tunesi, Diego Albani, Carmen Giordano

**Affiliations:** ^1^Department of Chemistry, Materials and Chemical Engineering “G. Natta”, Politecnico di Milano, Milan, Italy; ^2^Department of Neuroscience, Istituto di Ricerche Farmacologiche Mario Negri IRCCS, Milan, Italy

**Keywords:** microbiota-gut-brain axis, neurodegeneration, gut microbiota, microfluidic device, organ-on-a-chip, 3D culture

## Abstract

The steady increase in life-expectancy of world population, coupled to many genetic and environmental factors (for instance, pre- and post-natal exposures to environmental neurotoxins), predispose to the onset of neurodegenerative diseases, whose prevalence is expected to increase dramatically in the next years. Recent studies have proposed links between the gut microbiota and neurodegenerative disorders such as Alzheimer's and Parkinson's diseases. Human body is a complex structure where bacterial and human cells are almost equal in numbers, and most microbes are metabolically active in the gut, where they potentially influence other target organs, including the brain. The role of gut microbiota in the development and pathophysiology of the human brain is an area of growing interest for the scientific community. Several microbial-derived neurochemicals involved in the gut-microbiota-brain crosstalk seem implicated in the biological and physiological basis of neurodevelopment and neurodegeneration. Evidence supporting these connections has come from model systems, but there are still unsolved issues due to several limitations of available research tools. New technologies are recently born to help understanding the causative role of gut microbes in neurodegeneration. This review aims to make an overview of recent advances in the study of the microbiota-gut-brain axis in the field of neurodegenerative disorders by: (a) identifying specific microbial pathological signaling pathways; (b) characterizing new, advanced engineered tools to study the interactions between human cells and gut bacteria.

## Introduction

At the beginning of 2018, Pfizer announced its intention to end neuroscience research and development activity, including the effort on Alzheimer's (AD) and Parkinson's (PD) diseases, to re-allocate funding to those areas where it could provide the greatest impact for patients (Stimulus package, 2018). However, neurodegenerative disorders represent about one third of all Years Lived with Disability (YLDs) (Schroeder and Bäckhed, 2016) and between 2011 and 2030 an economic loss in the order of US$ 16.3 trillion worldwide is expected because of such debilitating conditions (Vigo et al., 2016).

Despite advances in basic research, we have not understood the complexity of the pathophysiology of the brain yet. This is apparent when considering that about 99% of clinical trials for new drugs against AD failed in the past 15 years (Cummings et al., 2014). However, we have some positive findings. For instance, the observation that, in China, AD has a low incidence among the elderly who regularly eat brown algae, has inspired the development of GV-971 (sodium oligomannate) molecule for the treatment of mild to moderate AD. *In vivo* studies have shown that an alteration in the composition of intestinal microbiota leads to the peripheral accumulation of phenylalanine and isoleucine, which stimulate the proliferation of Th1 proinflammatory cells. Phenylalanine and isoleucine can reach the brain and contribute to the neuroinflammation associated with AD. GV-971 molecule reduces the accumulation of phenylalanine and isoleucine and improves the health status of intestinal microbiota (Wang et al., 2019).

Another reason explaining the difficulties of developing effective therapies against neurodegeneration lies in the fact that neurodegenerative disorders have an overlapping symptomatology and they can be very hard to diagnose promptly. Currently, we lack adequate biomarkers and reliable models to gain some hints into what is happening inside the body. A breakthrough could come from recent studies on the interconnection between intestinal microbiota and neurodegeneration. They are expanding the role of gut microbiota (i.e., set of microorganisms living in the gastrointestinal tract, GI) in human physiology and throwing a spotlight on their pivotal importance in disease onset or progression.

Our current gut microbial consortia is the result of a long-lasting co-evolution with the host, which offers an ideal growing environment for many types of microorganisms. In turn, they provide a number of utilities for humans (e.g., protection against pathogens, digestion and assimilation of nutrients, regulation of immune system; Lynch and Pedersen, 2016; Shapira, 2016). To understand how deeply these guests can influence our body and change our state of health, we should consider that two individuals are equal for about 99.9% of the host genome, whereas they can differ for about 80–90% of the microbiome (Ursell et al., 2012). Recently, researchers have shown a possible correlation between quantitative and qualitative alterations in gut microbiota and several brain pathological conditions (e.g., autism, hepatic encephalopathy, epilepsy, AD) (Wu et al., 2016; Mancini et al., 2017; Strati et al., 2017; Szablewski, 2018). Therefore, the composition of gut microbiota could be an indicator of the health condition of the brain (Long-Smith et al., 2020). Currently, we are assisting to a growing recognition within the scientific and medical communities, of the link between gut microbiota and the CNS, known as the “microbiota-gut-brain axis.” The identification of the existence of this bidirectional communication has opened new scenarios in the study and understanding of brain diseases, also in the field of chronic neurodegeneration.

## Microbial Metabolites and Their Influence on Brain Pathology

**Figure 1:**
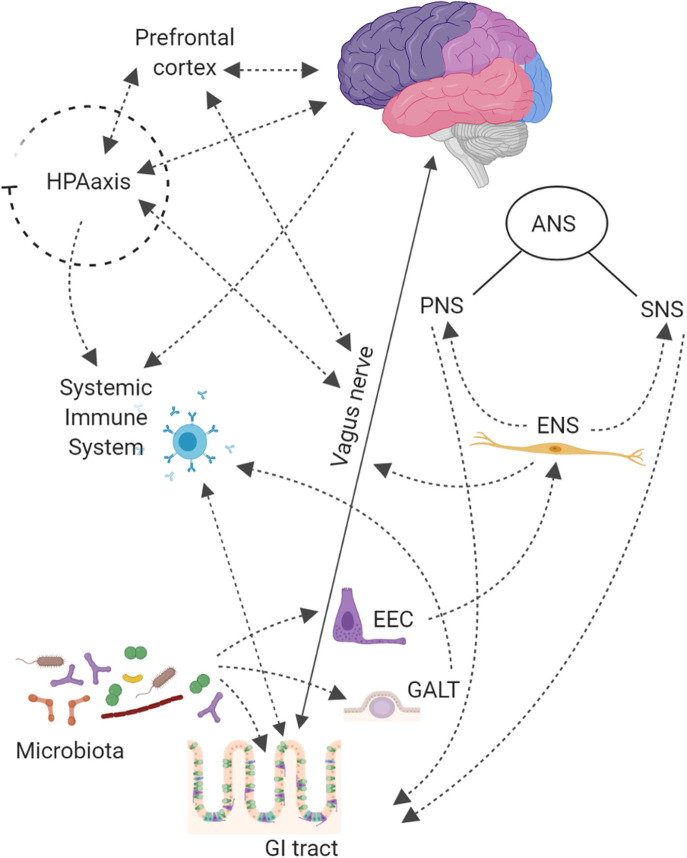
The Central Nervous System (CNS) is connected with the gut thought Autonomic Nervous System (ANS), that is divided into: Sympathetic (SNS), Parasympathetic (PNS), and Enteric (ENS) Nervous Systems. The SNS exerts mainly an inhibitory effect on GI tract and regulates GI blood flow. The PNS exerts both excitatory and inhibitory control over gastric and intestinal tone and motility. The main component of PNS is the Vagal Nerve that affects the peristalsis and the sphincter muscles of gut, and by afferent spinal and vagal sensory neurons. It sends feedbacks from the GI tract to the brain stem, which in turn engages the hypothalamus and limbic system for data processing. Finally, the ENS, located in the walls of the GI tract and described as a “second brain” (an extensive system composed of about 500 million neurons, exceeding those at the level of the spinal cord), communicates with the CNS via SNS and PNS. The ENS displays sophisticated coordination and exhibits plasticity and learning in response to changing dietary habits. In the intestine up to 80,000 vagal afferent nerve terminations are present, which send and receive signals, to and from the brain in a rate of 9:1. The Hypothalamic-Pituitary-Adrenal (HPA) axis coordinates adaptive responses to stress and activate memory and emotional centers in the limbic system of the brain and is also able to influence the composition of the gut microbiota and increase GI permeability. In the case of pathogen invasion, tissue damage, or homeostatic dysregulation, signals are processed by the CNS and sent to sympathetic and efferent vagal nerves, that release catecholamine and acetylcholine, respectively, under control of immune cells. The HPA axis through release of corticosteroids, with anti-inflammatory and immunosuppressive effects, regulates brain–immune pathway. The Gut-Associated Lymphoid Tissue (GALT) comprises 70% of the body's immune system; chronic stress has been shown to alter intestinal permeability (i.e., leaky gut syndrome), which is associated with a low-grade inflammation that can be functionally linked to several neurological disorders. On the other hand, also gut microbiota contributes to connect the gut to the brain compartment. Bacterial products can stimulate enteroendocrine cells (EEC) to produce several neuropeptides that can enter the bloodstream and/or directly influence the ENS, activate immune cells that release cytokines or activate the vagal nerve. This can affect the physiology of the brain, including neurotransmission and neurogenesis, but may also be involved in neuroinflammation.

The microbiota-gut-brain crosstalk has revealed a complex network system based on (i) vagus or enteric nervous, (ii) endocrine, (iii) immune, and (iv) humoral-mediated mechanisms (Figure 1; Carabotti et al., 2015; Bonaz et al., 2018). How microbiota signals can reach the brain is still debated.

Several studies have shown that the intestinal microbiota sends and receives a multiplicity of signals to and from the brain. These signals can modulate a variety of GI functions. It has become increasingly evident that microbe-related bidirectional gut-brain signaling plays a role in human cognitive functions, brain development and brain pathophysiology (Collins and Bercik, 2009). Diet has an important role in shaping the gut microbiota (Tables 1A–D) and the flux of its neurochemicals. The major metabolites secreted in the almost terminal GI tract (colon) by bacterial-mediated carbohydrate fermentation are the short-chain fatty acids (SCFA) (mainly butyric, propionic and acetic acids), that are able to trigger nerve-cell relevant responses, for instance by stimulating the sympathetic nervous system (SNS) and promoting the mucosal release of serotonin (Carabotti et al., 2015). SCFA also seem to interfere with pathological mechanisms important for AD, as the formation of soluble amyloid beta (Aβ) aggregates—associated with neurotoxicity and synaptic dysfunction—and therefore they could be of relevance for the development of next-generation probiotics that might help to promote resilience to neurodegenerative diseases (Ho et al., 2018). Moreover, some prebiotic fibers [e.g., inulin and fructooligosaccharides (FOS)] promote the growth of *Clostridium, Eubacterium, Fusobacterium, Roseburia*, and *Faecalibacterium* genera (Fu et al., 2019). These bacteria are important members of the commensal gut microbiota and produce high amounts of butyrate, that can pass the blood-brain barrier (BBB) and exhibits neuroprotective, cognitive and anti-depressive effects (Dalile et al., 2019). The gut microbiota can also stimulate enteroendocrine cells to produce several neuropeptides, such as peptide YY (PYY), NeuroPeptide Y (NPY), cholecystokinin (CCK), glucagon-like peptide-1 (GLP-1) and−2 (GLP-2), and substance P. They can enter the bloodstream and/or directly influence the enteric nervous system (ENS). They can affect neurotransmission and neurogenesis, but also neuroinflammation (Cani et al., 2013). Moreover, some bacterial species can produce other important brain-bioactive compounds, such as folate, serotonin (5-hydroxytryptamine, 5- HT), dopamine and γ-aminobutyric acid (GABA) (Asano et al., 2012; Selhub et al., 2014).

**Table 1A:** **(A–D)** Overview of communication pathways in the microbiota-gut-brain axis and the main effects on the GI tract and the brain. Neuronal communication pathway.

**Microbiota-gut-brain communication routes**		**GI and Brain effects**	**References**
A. Autonomic Nervous System (ANS)	A1. Sympathetic Nervous System (SNS)	A1.1. - Effect (e.g., mucosal secretions)	Borst et al., 2007; Watson et al., 2010; Browning and Travagli, 2014; Furness, 2016; Breit et al., 2018
	A2. Parasympathetic Nervous System (PNS, Vagus Nerve)	A2.1. ± Control on GI tone and Motility A2.2 Transmission of signals to and from the brain at a ratio of 9:1 A2.3 Expression of chemical and mechanosensitive receptors	
	A3. Enteric Nervous System (ENS)	A3.1 Control of gut functioning independently from SNS and PNS, but communication with the SNC through the ANS	
B. Hypothalamic Pituitary Adrenal (HPA) axis	B1. Corticotropin-Releasing Factor (CRF)	B1.1 Influence on gutphysiology	Farzi et al., 2018
	B2. AdrenoCorticoTropic Hormone (ACTH)	B2.1 Alteration of microbiome composition	

**Table 1B:** Endocrine communication pathway.

**Microbiota-gut-brain communication routes**		**GI and Brain effects**	**References**
A. Enteroendocrine cells (EEC)	A1. Ghrelin Cholecystokinin (CCK) Glucagon-like peptide 1 (GLP-1) Peptide YY (PYY) Serotonin (5-HT) → ENS	A1.1 Regulation of appetite, digestive processes, inflammation and neurological functions	Guilloteau et al., 2006; Berthoud, 2008; Bauer et al., 2016; Westfall et al., 2017; Breit et al., 2018; Farzi et al., 2018
B. Directly from the gut to the bloodstream	B1. Lipopolysaccharide (LPS), Neurotoxins, SCFA	B1.1 ± Systemic immune system, affect the blood brain barrier (BBB), and brain functions	Carabotti et al., 2015; Ho et al., 2018

**Table 1C:** Immune communication pathway.

**Microbiota-gut-brain communication routes**		**GI and Brain effects**	**References**
A. HPA axis	A1. ↑↓ pro-anti-inflammatory cytokines	A1.1 ↑↓ Intestinal and BBB permeability, ↑↓ systemic, and central inflammation	Dantzer, 2018
B. Gut-Associated Lymphoid Tissue (GALT)	B1. LPS, Neurotoxins, SCFA	B1.1 ↑↓ Intestinal and BBB permeability, ↑↓ systemic and central inflammation	Farzi et al., 2018

**Table 1D:** Humoral communication pathway.

**Microbiota-gut-brain communication routes**		**GI and Brain effects**	**References**
A. SNS	A1. Catecholamine (Noradrenaline)	A1.1 Alteration of growth, motility and virulence of pathogenic and commensal bacteria	Moreira et al., 2016
B. HPA axis	B1. Corticosteroids → anti-inflammatory and immunosuppressive actions	B1.1 Regulation of brain-immune pathway	Dantzer, 2018
C. Microbial-derived metabolites	C1. SCFA	C1.1 Stimulation of SNS, release of mucosal 5-HT, influence of memory and learning processes, ↓ Aβ aggregates and neuroprotective, cognitive, and anti-depressive effects	Carabotti et al., 2015; Ho et al., 2018; Dalile et al., 2019; Fu et al., 2019
	C2. Neuropeptides (PYY, NPY, CCK, GLP-1, GLP-2, and substance P)	C2.1 Influence on the ENS and effect on brain (patho)physiology	Cani et al., 2013
	C3. Folate	C3.1 Precursor in the brain biosynthesis of monoamine neurotransmitters (5-HT, epinephrine, and dopamine), implication in cognitive performances by reducing inflammation and homocysteine levels, support to the structure and functions of juvenile brain cells	Miller, 2008; Rossi et al., 2011; Oikonomidi et al., 2016; Ma et al., 2019; Naninck et al., 2019
	C4. 5-HT	C4.1 Immunomodulation, regulation of GI motility, secretion and sensation	Desbonnet et al., 2008; Kashyap et al., 2013; Oh et al., 2015; Reigstad et al., 2015
	C5. Dopamine	C5.1 Neurotransmitter involved in reward, motivation, memory, attention, and regulation of body movements, correlated with neurodegenerative disorders	Tsavkelova et al., 2000; Asano et al., 2012; Evrensel and Ceylan, 2015; Maiti et al., 2017
	C6. γ-aminobutyric acid (GABA)	C6.1 Neurotransmitter implicated in anxiety and depression	Schousboe and Waagepetersen, 2007; Bravo et al., 2011; Asano et al., 2012; Barrett et al., 2012; Ko et al., 2013; Selhub et al., 2014

Folate is the naturally occurring form of vitamin B9, mainly derived from dietary sources (e.g., leafy vegetables). Before entering the bloodstream, the digestive system converts folate into the biologically active form of vitamin B9, 5-methyltetrahydrofolate (5-MTHF), which is critical to produce monoamine neurotransmitters in the brain, such as serotonin, epinephrine, and dopamine (Miller, 2008). Folate is involved in cognitive performance by different mechanisms, such as reducing inflammation and homocysteine level. Chronic inflammation and elevated level of homocysteine are linked to neurodegeneration, but probably the most relevant implication of folate on the brain is its ability to modulate gene expression by an on/off mechanism. In particular, the low bioavailability of folate switches on the genes involved in the synthesis of Aβ (Oikonomidi et al., 2016; Ma et al., 2019). Folate also supports the structure and functions of juvenile brain cells (Naninck et al., 2019). In addition to the diet, probiotic bacteria in the human GI tract can synthesize B-vitamins. Lactobacillus (e.g., *L. casei, L. salivarius, L. plantarum*, and *L. reuteri*), Bifidobacterium (e.g., *B. bifidum* and *B. infantis*) and *Lactococcus lactis* and *Streptococcus thermophilus* are recognized to be folate-producing bacteria (Rossi et al., 2011).

Since the early 1950s, 5-HT has been the subject of intense biological research both for its activities as a neurotransmitter but also as an immunomodulator in the body periphery. 5-HT is also involved in the regulation of GI motility, secretion and sensation, and it has served as a basis for the development of novel treatments for GI disorders, such as Irritable Bowel Syndrome (IBS). Approximately 95% of serotonin is produced from tryptophan (TRP) by enterochromaffin cells in the gut, where it has a local neuromodulatory action (Kashyap et al., 2013; Oh et al., 2015). The gut microbiota influences serotonin levels, as reported by a study in germ-free (GF) mice, where a significantly lower plasma concentrations of 5-HT was measured compared to conventional mice (Wikoff et al., 2009). In addition, the oral ingestion of the probiotic *Bifidobacterium infantis 35624*, increased the levels of the serotonin precursor, TRP, in the plasma of rats, suggesting that this bacterial strain may have potential modulatory activities on TRP metabolism in the gut and could possibly be investigated as a novel therapy for depression (Desbonnet et al., 2008). However, the ability to produce 5-HT or induce its production does not seem to be restricted to Bifidobacteria and Lactobacilli, since strains of *Candida, Streptococcus, Escherichia*, and *Enterococcus* are also able to produce serotonin (Lyte, 2011). As mentioned, intestinal microbiota can modulate 5-HT levels by producing SCFA (particularly butyrate and acetate), which significantly affect Tryptophan hydroxylase 1 (TPH1) expression in a concentration-dependent manner (Reigstad et al., 2015). Further studies are needed to explore and evaluate the regulation of TRP metabolism by gut microbiota.

Dopamine is a neurotransmitter involved in reward, motivation, memory, attention and even regulation of body movements. It is now well-stated that low levels of dopamine in the brain are directly correlated with neurodegenerative disorders such as PD (Maiti et al., 2017). The biosynthesis of dopamine occurs both in neurons and peripheral compartments, including the GI tract. Tyrosine decarboxylase (TDC), whose genes are mainly encoded in the genome of *Lactobacillus* and *Enterococcus*, is essential for tyramine biosynthesis. Given the similarity between the chemical structure of L-tyrosine (precursor of tyramine) and levodopa (precursor of dopamine), this enzyme could also be involved in the synthesis of dopamine (van Kessel et al., 2019). High levels of dopamine have also been found in the gut lumen of specific pathogen-free (SPF) mice compared to GF mice (Asano et al., 2012). Moreover, Bacillus (e.g., *B cereus, B. mycoides* and *B. subtilis*) and Serratia (e.g., *S. marcescens, S. aureus*), as well as *Proteus vulgaris*, and *Escherichia coli*, have shown capacity to produce dopamine (Tsavkelova et al., 2000; Evrensel and Ceylan, 2015).

GABA is the main brain inhibitory neurotransmitter. It regulates many physiological and psychological processes. Anxiety and depression involve dysfunctions in the GABA system (Schousboe and Waagepetersen, 2007). Some strains of *Lactobacillus* and *Bifidobacterium* produce GABA from monosodium glutamate and can influence the gut-brain axis (Yunes et al., 2016). A screening of several gut bacteria has identified *Lactobacillus brevis* and *Bifidobacterium dentium* as the most efficient GABA producing species (Barrett et al., 2012). Another GABA producer tested in rats, *Lactobacillus brevis FPA3709*, has an effect similar to Fluoxetine (a common antidepressant drug), but without the drug side effects, which include loss of appetite and body weight (Ko et al., 2013). At the level of gene expression, the ingestion of *Lactobacillus rhamnosus JB-1*, was shown to alter the mRNA expression of both GABA A and B receptors, which have been implicated in anxiety and depression (Bravo et al., 2011). Strains of both *Lactobacillus* and *Bifidobacteria* genera are commonly used as probiotics and are present in many traditional fermented foods, especially dairy products (Selhub et al., 2014).

## Pathological Conditions of the Microbiota-Gut-Brain Axis: Focus on Alzheimer's and Parkinson's Diseases

A causal relationship between alterations in the quality and/or quantity of individual microorganisms residing in the GI (known as dysbiosis) and a pathological outcome in the brain structure and functions is far from being revealed. Despite this, several interconnecting pathways may link these anatomical compartments and lead to their crosstalk and dysfunctions. To this respect, immune-mediated mechanisms play a pivotal role. Several studies have highlighted that an increase in intestinal permeability can allow the translocation from the gut to the systemic circulation of either microorganisms or their components (e.g., lipopolysaccharide—LPS, the major outer membrane component of Gram-negative bacteria) and metabolites (e.g., β-N-methylamino-L-alanine—BMAA, a neurotoxin produced by Cyanobacteria or blue-green algae). They contribute to the triggering of systemic inflammation and release of pro-inflammatory cytokines, which may induce brain damages via direct or indirect effects (Köhler et al., 2016; de la Fuente-Nunez et al., 2018). A new line of investigation has suggested that some species of Cyanobacteria, present in small numbers in the GI tract, are potentially responsible to induce neurodegeneration through the production of BMAA (Burton, 2013).

BMAA has been found in significant concentrations in the brains of AD, PD and amyotrophic lateral sclerosis (ALS) patients (Brenner, 2013). It is an excitotoxin that activates the metabotropic glutamate receptor 5, ultimately leading to increased oxidative stress (Ballatori et al., 2009). As a result, neurons and glial cells are unable to effectively control the production of reactive oxygen species (ROS) and reactive nitrogen species (RNS) in the brain. Recently, Tan et al. have developed a new antibody to analyse the extent, propagation, inter-cellular dynamics, subcellular locations, and target cells of BMAA accumulation. They have discovered that BMAA-mediated toxicity is due both to a direct mechanism exerting a neurotoxic action on motor neurons and an indirect mechanism exploiting the activation and/or induction of dysfunctions of glial cells, resulting in an amplification of the neurotoxicity (Tan et al., 2018).

BMAA-associated toxicity is just one possible causal link in the field of the microbiota-gut-brain axis and neurodegenerative disorders. We have other evidence connecting molecular players of AD and PD and gut microbiota. Clumped, misfolded proteins and brain inflammation are common features of AD and PD and both lead to neuronal damage. In AD, the pathological hallmarks are abnormal structures formation called Aβ plaques and neurofibrillary tangles which are thought to contribute to the death of the neurons in the brain (Eckerström et al., 2018). It is possible that microbes contribute to this process. The term “mapranosis” (Microbiota Associated Proteopathy And Neuroinflammation + osis) has been coined for the process by which amyloid proteins produced by microbes, such as *Streptococcus, Staphylococcus, Salmonella, Mycobacteria, Klebsiella, Citrobacter*, and *Bacillus* genera, can prime brain Aβ misfolded structure and enhance inflammation in the nervous system (Kujala et al., 2011; Sharon et al., 2016; Uesaka et al., 2016). These gut bacteria can migrate to the systemic circulation, and then reach the brain, taking advantage of transient pathological conditions characterized by an increased permeability of the BBB and reduced immune response. Another interesting theory linking amyloid misfolding and microbes has shown that Aβ can protect against yeast and bacterial infections in both animal models and human cell cultures. The Amyloid Precursor Protein (APP) is processed by specific proteases called α, β, and γ- secretase in different peptides, including Aβ. Aβ would seem to have antimicrobial properties both *in vivo* and *in vitro* experiments. During their oligomerization, Aβ peptides are able to trap invading microorganisms in the plaques, including bacteria (e.g., *Salmonella enterica*), fungi (e.g., *Candida albicans*), viruses (e.g., *Herpes simplex*), and parasites (e.g., *Toxoplasma gondii*) (Kumar et al., 2016). This activity suggests that Aβ could belong to anti-microbial peptides (AMPs), primary effector molecules of innate immunity. Nevertheless, the uncontrolled or chronic formation of Aβ plaques in response to these infections could cause neuroinflammation and neurodegeneration (Stilling and Cryan, 2016). Interesting, APP/PS1 mice, a model of AD, showed a reduced accumulation of microglia and astrocytes around Aβ plaques in the hippocampus after antibiotic treatment. However, it is not completely clear whether this effect depends on microbe depletion or a secondary anti-inflammatory action of the antibiotic itself (Harach et al., 2017). Further evidence linking AD, amyloid deposition and gut microbiota comes from APPSWE/PS1ΔE9 mouse model of Aβ amyloidosis, where gut microbiota influenced the deposition of Aβ plaques by the modulation of neuroinflammation (Minter et al., 2016).

In the CNS neuroinflammatory responses are complex and mediated by (a) central and (b) peripheral immune components. Toll-like receptors (TLRs) are important players of innate immunity, able to recognize microbes by binding to pathogen-associated molecular patterns. They also bind soluble Aβ, detected by TLRs expressed by microglia, triggering the activation of inflammasome complexes and leading to neuroinflammatory responses. Microglia cells are a population of glial cells dealing with the first and primary active immune response in the CNS. To assess the role of intestinal microbiota on this cell type, several studies have exploited animal models (e.g., GF mice) and drugs (e.g., antibiotics). What has emerged is that unbalanced intestinal microbiota can change the expression profiles of inflammatory genes and influence the basal surveillance state of microglia cells, modify cell morphology and reduce inflammatory responses against pathogens (Minter et al., 2016). Nevertheless, the mechanisms controlling microglial clearance of Aβ from the brain are still unclear (Manchikalapudi et al., 2019).

Microbes can contribute to the inflammatory state of the host by producing the so-called microbial-associated molecular patterns (MAMPs), which gut commensal microbes express to communicate with the immune system. They can be sensed by pattern recognition receptors (PRRs) (e.g., TLRs) located on immune, epithelial and endothelial cells. This association induces the production of cytokines (e.g., type I interferons (IFNs), tumor necrosis factor (TNFα), interleukin 1 and 6 (IL-1, IL-6) and then differentiation of effector T-cells, thus activating and attracting innate immune cells. Moreover, circadian clock seems to regulate the expression levels of TLRs on intestinal epithelial cells (IECs) (Alegre et al., 2014). All these microbial signal molecules may impact on neuroinflammation and ultimately on AD pathology (Cattaneo et al., 2016).

Another important microbial-derived molecule that can contribute to brain neuroinflammation by TLR activation is Curli protein, which shares structural and biophysical properties with amyloids (Chapman, 2002). It is produced by *Escherichia coli*, which secrete an extracellular fiber called curli, able to help bacterial cells to bind together and form a biofilm to resist physical or immune agents (O'Toole et al., 2000). It is composed of two proteins (CsgA and CsgB), which are directed to the outside of the cell by a dedicated export system (Van Gerven et al., 2015). The curli fiber secreted by *E. coli* share structural and biophysical properties with amyloids (Chapman, 2002). *Escherichia coli* curli fiber can stimulate the innate immune system by recognizing the TLR2–TLR1 heterocomplex (Tükel et al., 2009, 2010).

The relationship between gut microbiota and AD has been explored also in patients. A recent clinical study has reported an altered gut microbiota composition in AD cases, with an increased prevalence of Firmicutes and Actinobacteria, and a decrease of Bacteroidetes in comparison to a control group. The same study has also reported a correlation between the relative abundance of fecal bacterial taxa at the genera level and pathological biomarkers in the cerebrospinal fluid (CSF) of patients. Specifically, the Authors have focused on Aβ_42_/Aβ_40_ ratio in the CSF. It decreases in AD and provides information on the burden of pathological Aβ in the brain. It has showed a positive correlation with the abundance of gut *Bifidobacterium*, suggesting that, conversely, a decrease in *Bifidobacterium* may correspond to a reduction of Aβ in the CSF and therefore to an increase at cerebral level (Vogt et al., 2017). Therefore, *Bifidobacterium* connotates as a protective microbial strain. In fact, *Bifidobacterium spp*. has an important role in maintaining the gut homeostasis, modulating the inflammatory response (e.g., reduction of IL-6 and TNF-α), reducing LPS release and preserving intestinal permeability that prevents pathogens translocation (Ruiz et al., 2017). The presence of LPS in either plaques or blood vessels has been confirmed in a post-mortem biopsy study of patients with AD. LPS has been found in the gray matter of the superior temporal gyrus and the frontal lobe of white matter commonly involved in this disease (Zhan et al., 2016). This could support the hypothesis of the role of LPS in the formation of amyloid plaques. LPS can affect the integrity of the BBB and induce a wide range of inflammatory responses in the brain, including increase in cytokines, chemokines, prostaglandins, and nitric oxide (NO), responsible of neuronal damage (Mallard, 2012; Wensink et al., 2016). Interestingly, high levels of histamine have been found in AD, which raise the levels of NO, increasing neurodegenerative processes (Alvarez et al., 1999). Some bacteria, such as *Lactobacillus, Lactococcus, Streptococcus, Pediococcus*, and *Enterococcus* spp., are able to produce histamine (Landete et al., 2008) and this could further explain how an altered intestinal microbiota can be related to AD.

Finally, it is worth to notice that not only gut microbiota may be causative for AD, but a completely different cross-talking mechanism may connect oral dysbiosis with the disease. In fact, oral microbiome of immunodepressed individuals embraces a range of abnormal bacterial phylotypes perpetuating a range of inflammatory conditions. This chronic condition may involve a wide range of extra-oral organs including the brain. Oral bacterial amyloids are produced by a wide range of resident microbes, including the common symbiont *Streptococcus mutans* (Oli et al., 2012). The dysbiotic microbial communities profiled in periodontitis reflect similar traits with AD microbiome. For example, the periodontal pathogen *Porphyromonas gingivalis* shows immunosuppressive properties by promoting interleukin-2 (IL-2) inhibition and, even if lacks the curli gene, it may trigger alternative inflammatory mechanisms that indirectly activate β-secretases and contribute to host-derived Aβ response. In fact, it produces proteases called gingipains [i.e., lysine-gingipain (Kgp), arginine-gingipain A (RgpA), and arginine-gingipain B (RgpB)], that are virulence factors useful for inactivating the host defenses and destroying tissues facilitating its colonization and nutrient acquisition for its growth. *P. gingivalis* could even access the brain via (i) monocyte infection and brain recruitment, (ii) inflammation of the BBB, and (iii) diffusion through the olfactory or trigeminal nerves in the brain (Dominy et al., 2019). Moreover, tau pathology observed in AD brains may be due to the transneuronal spread of *P. gingivalis*, with direct damage of the gingipain proteolysis on tau or indirectly activation of human proteases acting on tau. CSF analysis identified the presence of gingipain in AD brain, as well as the DNA of *P. gingivalis* and gingipain antigens (Dominy et al., 2019). Finally, there is a correlation between periodontitis and loss of mental function, placing periodontitis as a risk factor for AD. For this reason, preventative measures must include dental care as an intervention from an early age (Friedland, 2015).

The second most common degenerative neurological disorder is PD. This pathology is characterized by α-synuclein (α-syn) aggregation in insoluble inclusions inside neuronal body (known as Lewy bodies) and loss of dopaminergic neurons in the Substantia Nigra pars compacta (SNpc). α-syn is a small protein normally present at presynaptic level, where it is thought to promote the formation of the SNARE complex. When in soluble form, the protein can cross the BBB (Felice et al., 2016). Many studies highlight a connection between PD and GI dysfunctions, suggesting a possible involvement of the gut microbiota also in this neuropathology (Poirier et al., 2016). An extensive review summarizing microbiota-gut-brain axis and PD has been provided by others (Nair et al., 2018).

Just to summarize the most relevant findings in this field, low amount of GABA-producing gut bacteria (e.g., *Bifidobacterium*) have been observed in PD patients (Minato et al., 2017). In the CNS, the Ca^2+^/GABA signaling is relevant for neuronal activity and calcium homeostasis, therefore a decline in the GABA amount may contribute to neurodegeneration (Błaszczyk, 2016). Another hypothesis implicating a role of gut microbiota in PD regards microglia-mediated neuroinflammation through the (a) humoral and (b) vagal afferent pathways. In the humoral pathway, Gram-negative bacteria release endotoxins that can induce microglia activation and misfolding of α-syn (Huang et al., 2018). Otherwise, in the vagal afferent pathway an intriguing hypothesis states that α-syn is supposed to act like a prion protein that is abnormally deposited in the ENS of patients with PD and can also access the CNS through the vagal nerve itself. As for AD, gut microbiota is not the only possible player in triggering or modulating the disease. To this respect, a very relevant example is represented by *Helicobacter pylori*. Intragastric *H. pylori* infection influences motor function and interferes with levodopa absorption (an important PD drug treatment). It has been seen that levodopa absorption can improve up to 50% after *H. pylori* eradication therapy, while patients with PD may experience impaired motor function after failed eradication therapy (Huang et al., 2018).

Our knowledge of gut microbiota and PD is expanding also to clinical cases. A recent study on PD patients, has revealed an association between changes in gut microbiota composition and PD, accompanied by altered concentrations of SCFA. SCFA are the end products of fermentation of dietary fibers by the anaerobic intestinal microbiota and it have been shown to exert multiple beneficial effects on human body. Butyrate, one of the most important and studied SCFA, has been shown to have anti-inflammatory effects, to promote a physiological intestinal epithelial barrier and to interacts with the ENS, increasing colon contractility. Reduced butyrate concentrations in feces, consistent with the significant decrease in *Faecalibacterium prausnitzii* (a SCFA producer) in patients with PD, could have significant effects on ENS and contribute to the GI dysmotility typical of this disease (Unger et al., 2016). Finally, bacterial proliferation in the small intestine, known as SIBO, has also been proposed to be involved in motor impairments of PD patients and its eradication seems to improve motor fluctuations (Tan et al., 2014; Vizcarra et al., 2018).

Other studies reported that fecal microbiota of patients with PD showed increased *Enterobacteriaceae* and decreased *Prevotellaceae* compared to control group. *Prevotella* is a genus of Gram-negative bacteria involved in the breakdown of complex carbohydrates from which SCFAs and B group vitamins (e.g., thiamine and folate) are produced, promoting a healthy intestinal environment (Arumugam et al., 2011). Unless compensated by diet, a decrease in *Prevotella* may lead to essential vitamins reduction, a condition that has been reported in PD patients (Scheperjans et al., 2015). In addition, a decreased abundance of *Prevotella* and an increased abundance of *Lactobacillaceae* have been associated with lower concentrations of ghrelin. Ghrelin is a gut hormone that is involved in the maintenance and protection of normal nigrostriatal dopamine function (Andrews et al., 2009) and impaired ghrelin secretion has been reported in PD patients (Unger et al., 2010). Furthermore, intestinal biopsies of PD patients show marked differences in the sigmoid mucosa with significant reductions in anti-inflammatory butyrate-producing bacteria (e.g., *Roseburia* and *Faecalibacterium* spp.) and a clear increase in proinflammatory Proteobacteria species of the genus *Ralstonia* compared to healthy age-matched controls (Westfall et al., 2017). PD patients also exhibit an accumulation of α-syn neurons in the sigmoid mucosa 2–5 years before developing neurological symptoms of PD (Forsyth et al., 2011). Many studies report that PD patients, following the administration of probiotics such as *Lactobacillus casei Shirota* or others belonging to *Enterococci, Bifidobacteria* and yeasts, led to a significant improvement of GI symptoms, with abdominal pain reduction (Felice et al., 2016). From the above described picture, AD and PD etiopathology may have a causal link with gut microbiota and more generally with human microbiotas, even though more research in the field is mandatory.

The clinical studies that survey chronic disorders, as AD and PD, will need to consider the “personal microbiome” composition in the context of temporal variability, considering the ecological principles that shape and govern stability, since the production of microbial metabolites, as well as the relative abundance of microorganisms inhabiting the human body, greatly depends on diet, sex, life style, and microbial gene expression itself. The main challenges for an effective clinical intervention aimed at improving the host-microbe interactions are linked to the definition of safe and effective methods for the manipulation of the microbiota and its administration and monitoring over the course of experiment.

A final consideration is to underscore that AD and PD neurodegeneration is only one of the possible fields in brain pathologies where the microbiota(s) may have a role. In fact, many other human studies found in the literature addressing the role of gut microbiota in brain disorders are about the spectrum of behavior disorders, such as Attention-deficit/hyperactivity disorder (Jiang et al., 2018); stress-related disorders (Ghavami et al., 2018); and major depressive disorder (Lin et al., 2017; Chen et al., 2018). The approaches used to investigate possible connections between gut and brain include (i) gut microbial manipulation with antibiotics (Schmidt et al., 2018); (ii) pre/probiotics administration (Tillisch et al., 2013; Chambers et al., 2015); (iii) fecal microbiota transplantation (Jalanka et al., 2018); (iv) post-mortem examination of the brain (Zhan et al., 2016); (v) metabolomics/metagenomics analysis of biological samples (e.g., feces, mucosal biopsy) (Evangelisti et al., 2017; Labus et al., 2017).

## Experimental Models To Address the Role of Gut Microbiota in Brain Neurodegeneration

In the last decade, the study of neurodegenerative diseases (e.g., AD and PD) has proved that, the idiopathic form of these diseases is clinically and neuropathologically like their rare familial forms. These phenotypic similarities have driven the development of a vast array of genetically modified cell and animal models, based on descriptive mutations for each pathology (Duty and Jenner, 2011). The studies conducted in experimental models have provided invaluable information on the pathogenesis and pathophysiology of these conditions, and *in vivo* models may be a tool also for unraveling the role of gut microbiota in AD/PD.

In fact, the role of the host gut microbiota on the physiology and functions of the CNS is largely investigated by many animal manipulations, including genetic (e.g., gene knockouts; Götz et al., 2004, 2018), gut microbiota composition (e.g., GF or gnotobiotic mice), antibiotic treatment (Kennedy et al., 2018), dietary interventions (e.g., pre/pro or post-biotic administration; Maguire and Maguire, 2019) and fecal transplantations (e.g., human microbiota-associated (HMA) mice; von Klitzing et al., 2017). However, animal and human GI tracts do show differences, also deriving from diet style, that leads to divergences in gut microbiota composition and crosstalk with the host, making a direct translation to human less direct (Nguyen et al., 2015; Arrieta et al., 2016). Beyond these limitations, the 3Rs rule (i.e., reduction, refinement, and replacement of animal testing) is increasingly encouraged, pushing forward the development of advanced *in vitro* approaches to investigate neurodegenerative diseases (Raimondi et al., 2019). In the following paragraphs, we are going to present innovative solutions for microbiota *in vitro* culturing, a mandatory step for the assessment of its pathological impact on brain tissue.

### Continuous Culture Systems for Gut Microbiota

Laboratory-scale continuous culture systems are the most used and studied technology to simulate the spatial, temporal, and environmental features that microbes experience within the GI environment. Depending on the model, single or multiple batch vessels are inoculated with fresh fecal samples and cultured under specific O_2_ concentration, temperature, pH, and feed medium conditions. Being host-free systems, these models are ideal systems to study microbial perturbations from exogenous stimuli, as microbial changes (e.g., relative abundance, α-β diversity and metabolism) can be measured without any concurrent effect on the host. Currently, the most advanced systems are: simulator of the human intestinal microbial ecosystem (SHIME), host-microbiota interaction module (HMI), TNO gastro-intestinal model (TIM) and dynamic gastro-intestinal simulator (SIMGI) (von Martels et al., 2017).

#### Simulator of the Human Intestinal Microbial Ecosystem (SHIME)

The SHIME is composed of a series of five interconnected reactors representing the stomach, the small intestine, the ascending, the transverse, and the descending colon, respectively. Peristaltic pumps provide a constant flow rate of fecal microbiota-enriched medium along the system, while maintaining a constant temperature of 37°C by means of a custom temperature controller. The stomach and the small intestine reactors are supplied three times a day with a nutritional medium and pancreatic juice, respectively. Then, the output of the second reactor is pumped downstream to the following colon reactors. Here, the media are homogenized by magnetic stir bars and the pH values are tuned accordingly to the GI tract modeled (e.g., stomach-2.0, small intestine-7.0, ascending colon-between 5.6 and 5.9, transverse, and descending colon-between 6.6 and 6.9), while monitoring constantly their volumes. The possibility to control the flow rates in the first two reactors and control the volumes in the last three ones, allowed modeling the physiological parameter of retention times. The SHIME was kept in anaerobic condition by flushing the reactors with nitrogen or a gas mixture (90% N_2_ and 10% CO_2_) while maintaining microbiome stability over a long timeframe (2–4 weeks) with the possibility to monitor microbiome adaptation to stimuli (Molly et al., 1993; Van de Wiele et al., 2015). This system is widely accepted as the main reference in the field of human GI models, due to its appreciable advantages, such as the modularity of each tract, and its completeness. Moreover, the possibility to use two SHIMEs in parallel (TWIN-SHIME configuration) opens to the possibility to investigate the inter-individual variability of gut microbiota behavior, under different treatments in a single experiment (Van de Wiele et al., 2015). However, the SHIME lacks a reactor specifically dedicated to the modeling of nutrient absorption, such as cells monolayers, because it simply transports the nutrients along the system. Moreover, the flow of fluids does not reproduce the peristaltic movements of the intestine, which are fundamental for the bacteria clearance in the gut and adsorption of molecules *in vivo*. Although this system is suitable for the study of the behavior of the gut microbiota, it does not take into account the presence of mucus which is the complex physiological environment where bacteria live and diffusion of substances is regulated (Sardelli et al., 2019). In order to improve the SHIME to overcome this last limitation, the same group optimized the system including the mucosal component in the colon compartments (M-SHIME). This further improvement allowed the gut microbiota to adhere to mucus layer and study the differentiation between mucosal and luminal microbiome (Van de Wiele et al., 2015).

#### Host-Microbial interaction Module (HMI)

The HMI module is a custom-made co-culture system composed of two divisions, named luminal and host compartments, and perfused with a semi-continuous flow of culture media. The first compartment contains intestinal microorganisms, while the second hosts enterocyte-like cells (e.g., Caco-2). A functional double layer of mucus/semi-permeable membrane between cells and microorganisms and an artificially mucus layer above the intestinal cells complete the system. The HMI module can be connected to an adapted version of the SHIME, containing only three reactors (i.e., stomach, small intestine and ascending colon). The small intestine reactor is then inoculated with a fresh fecal sample and, after passing the adapted SHIME reactors, the effluent flows through the luminal compartment of the HMI module. The host compartment, reproducing intestinal epithelial barrier, receives a semi-continuous flow of culture medium in the opposite direction. In the HMI module, the host cells are viable for ~48 h and the exposure to the complex microbial community or a selected microorganism allow to study the host-microbiome interactions (Possemiers et al., 2013; Marzorati et al., 2014).

The inclusion of cells and semi-continuous flow of media were the main improvements of this system with reference to the SHIME. Indeed, these features allow to study the adhesion of bacteria and their influence on the epithelial barrier in a single experimental set-up, allowing to unveil the diffusion-related mechanism behind the microbiota and host crosstalk. Moreover, the possibility to include the HMI downstream the SHIME opens the access to the investigation and validation of forefront therapeutic strategies, such as the use of probiotic and prebiotic.

#### TNO Gastro-Intestinal Model (TIM)

The TIM is a predictive *in vitro* simulation of the whole digestive system fabricated through a combination of engineering, chemicals, bacteria and dedicated software. It is divided in two sub-systems. The first one is the TIM-1, composed of four compartments that simulate the stomach, duodenum, jejunum and ileum and are connected by peristaltic valve pumps with both mixing and pushing purposes. The set-up allows the movement of a precise amount of chime, while two dialysis systems filter the compounds, thus reproducing the physiological process of absorption. Indeed, the input meal is mixed with artificial saliva containing electrolytes and α-amylase and grinded with a food processor. Temperature and pH are constantly monitored and secretions for gastric (i.e., electrolytes, pepsin, and a fungal lipase) and duodenal (i.e., electrolytes, bile and pancreatin) compartments are instilled. Water-soluble products are removed by dialysis through membranes connected to jejunal and duodenal compartments, while lipophilic products are removed through a filter that passes micelles, but retains fat droplets.

In addition to the TIM-1, the TIM-2 simulates the large intestine, including its microbial component (Minekus, 2015).

TIM-2 works in an anaerobic condition, keeps the pH at 5.8 and is equipped with a dialysate system which prevents the accumulation of metabolites and maintains an active microbiota for up to 3 weeks. The dialysis system guarantees physiological concentrations of microbial metabolites in the lumen (e.g., SCFA). The microbiota is fed with Simulated Ileal Efflux Medium (SIEM) mimicking the components that reach the colon from the terminal ileum (Venema, 2015).

The TIM models have the advantages to accurately replicate the digestion of food, by mimicking both chemical reactions and physical loadings. However, TIM is not able to investigate the effect of the digestion of the meal on the intestinal barrier. Indeed, it lacks in modeling the cells of the intestine and the mucus, which had a pivotal role in both active and passive diffusion of the digested molecules toward the systemic circulation. Despite this, the TIM can be used in combination with static cell cultures, offering a preliminary tool to study also the host-microbiota interactions (Déat et al., 2009).

#### Dynamic Gastro-Intestinal Simulator (SIMGI)

SIMGI is a computer-controlled GI *in vitro* model that is designed to simulate continuously and simultaneously different physiological processes in the GI tract while hosting the colonic gut microbiota. This automated system includes models of the stomach, the small intestine and the large intestine. The stomach compartment is composed of two flexible walls, covered by a methacrylate jacket, where water is pumped to maintain a stable temperature (37°C) while allowing mixing of the contents through peristaltic movements. The gastric contents move into the small intestine reactor, where they are mixed with the intestinal secretions (pancreatic juices and bile) by magnetic stirrer and maintained under anaerobic conditions and controlled pH. The slurry continues toward the compartments of the large intestine (ascending, transverse and descending colon), where the gut microbiota from fecal inoculum is hosted in anaerobic conditions by means of N_2_ flushing, controlled pH and continuous stirring. The large intestine compartment is made up of three double-jacket glass reactors, maintained at different pH for the ascending (5.6), transverse (6.3), and descending (6.8) colon through an acid/base pumping system. SIMGI software allows to study the digestion of selected substrates in the stomach, the small intestine and by the colonic microbial community. Moreover diet-induced changes on the gut microbiota and its metabolic activity can be monitored (Barroso et al., 2015). Even though the concept of the SIMGI may be apparently similar to other models, in particular the SHIME and TIM, it has some further improvements. For example, it allows the contemporary modeling of both the digestion and the absorption of the nutrients in the same system (differently than TIM). Moreover, the peristalsis in the stomach is obtained by peristaltic pumps and to stirring (like in SHIME) resembling more accurately the *in vivo* situation. Moreover, the precise pH-control allows the passages of materials from the stomach with defined acidification curve, which is a useful tool in the definition of microbiota survival in different environmental conditions, as well as pro and prebiotic availability after the passages in the upper GI tract.

### Miniaturized Research Systems to Model Gut Microbiota

Another strategy to study cell-microbe interactions addresses the use of miniaturized systems. These systems are the advanced versions of the permeable supports used for adhesion-dependent cell studies (i.e., Transwell), which were modified to reproduce highly diversified growth conditions, such as those required by the co-culture of gut microbiota and cells. In this view, the main challenge is to obtain coexistence of anaerobiosis and aerobic condition in the same system, which is a not-trivial goal. To date, the most advanced miniaturized technology aimed to simulate in *vitro* a whole organ or human compartment is the system so-called organ-on-a-chip (Sung, 2018). This revolutionary technology consists of one or more devices hosting 3D constructs that, due to suitable scaffolds (i.e., cross-linked natural-based polymeric materials with supporting elements) or hydrogel matrices, are capable to provide a more *in vivo*-*like* spatial and physical architecture for cell culture and induce their real physiological activity. Moreover, the presence of perfusion media inside the culture chambers of these systems guarantees both a physiological exchange of nutrients and metabolites between cells and medium and a shear stress that stimulates cell proliferation, growth and activity (Coluccio et al., 2019). As outlined above, miniaturization is also the key feature of this technology that exploits a very low amount of reagents and materials for the experiments. The integration with specific electrodes able to measure cell viability or sensors for the detection of parameters like pressure, pH, O_2_ concentration, temperature, etc. allows to have an integrate control system. We now focus on small-scale systems aimed at gut microbiota culturing, such as Transwell systems, HoxBan model, Gut-on-a-chip, Human-Microbial cross talk (HuMiX), Microfluidic cell culture device (μFCCD), Shim's microfluidic gut-on-a-chip and multiorgan-on-a-chip.

#### Transwell Co-culture System for Apical Anaerobic Barriers

To overcome the common limits of anaerobic configuration, Ulluwishewa and colleagues developed a novel Transwell co-culture system, a variant of the conventional Transwell systems. It is composed of an apical compartment with anaerobic environment, obtained sealing it from the outer environment hosting the microorganisms (e.g., *Faecalibacterium prausnitzii*) and a basolateral compartment separated by a permeable membrane in which cells that mimic intestinal epithelium (e.g., Caco-2 cells) are seeded and cultured to form a confluent and differentiated monolayer barrier between the two compartments (Ulluwishewa et al., 2015). This model allows an apical-anaerobic cell culture for around 12 h and a limited viability of microrganisms. The presence of two integrated electrodes, one in the basolateral chamber and one in the apical allowed the non-invasive measurements of the transepithelial electrical resistance (TEER) of the gut barrier every 30 min. A study with *Faecalibacterium prausnitzii* showed that supplementation with antioxidants (e.g., riboflavin) and acetate in the apical medium improved *F. Prausnitzii* growth under low oxygen levels (Khan et al., 2014). The results carried out with this kind of system are always partial since only a very short culturing time is possible and only one bacterial strain can be cultured in the model. In fact, although this system allowed putting in communication an aerobic environment with an anaerobic one, it had the strong limitation to lack the dynamic environment that is indispensable to run across long culture periods. Moreover, the lack of shear stress, impedes the formation of a suitable gut barrier, lacking of microvilli, with also the risk to expose bacteria to the oxic environment of the basal chamber. The hydraulic integration of the system with a chemostat for long time culture maintenance of bacteria and with a syringe pump to provide both perfusion of culture medium and shear stress on the intestinal cells would allow overcoming all of the above-mentioned limitations.

#### Human Oxygen Bacteria Anaerobic (HoxBan) Co-culturing System

The Human oxygen Bacteria anaerobic (HoxBan) co-culturing system is another system that aims to model the host-microbe interactions simulating the oxic-anoxic interface *in vitro*. The HoxBan consists of an anaerobic and aerobic compartment that are created into a sterile 50 mL polypropylene centrifuge tube. The bottom compartment contains the anaerobic bacteria within specific agarose culture medium (i.e., yeast extract, peptone, fatty acids, acetate, glucose, and 1% agar). The upper portion contains a Caco-2 monolayer grown on coverslips placed facing down and overlaid with cell culture medium. The agar from the top to the bottom ends of the 50 mL centrifuge tube creates a gradient of oxygen concentration, mimicking the physiological human intestinal epithelium environment. The obligate anaerobic bacteria are excluded from oxygen and can grow at the lower end of the gradient (Sadaghian Sadabad et al., 2016). Despite being a very simple model, it reproduces the interactions between the epithelium and microbiota, and gives the possibility to study the growth, the consumption/production of metabolites, and the behavior of human cells and bacteria, as well as their reciprocal interactions (von Martels et al., 2017). In this system, bacterial colonies of *F. Prausnitzii* continued to grow up to 36 h in co-culture with Caco-2 cells. Meanwhile, even after 24 h, the Caco-2 cells remained viable and continued to divide actively; moreover, since they do not have the ability to secrete mucus, it was artificially added to the cell monolayer (Sadaghian Sadabad et al., 2016). The HoxBan represents a very simple model to reproduce and not require any complex or custom-made set-up to create an oxic-anoxic interface. Unlike the previously described model, which exploits Transwell plates, the HoxBan system is not compatible with any TEER measurement system and with any optical system to observe cells and bacteria during the experiments. Again, the absence of a dynamic environment that lacks medium flux, usually provided by the presence of a chemostat and a system of syringe pumps, makes this model too simplistic to faithfully reproduce *in vivo* conditions. Improvements to this model are possible by using primary intestinal cells together with a much more complete population of human gut microbiota.

#### Gut-on-a-Chip

Gut-on-a-chip co-culture systems simulate the GI ecosystem to study host-microbiota interactions and inflammatory processes at mechanistic level. This model allows stable long-term (~2 weeks) co-culture of IECs and commensal microbes of the gut microbiota (Kim et al., 2012; von Martels et al., 2017). It is composed of two optically clear flexible polydimethylsiloxane (PDMS) microchannels, the lower one represents blood vessels and the upper one the gut lumen, separated by a porous flexible PDMS membrane coated by Extracellular Matrix (ECM) proteins and covered by Caco-2 cells. In addition to the low shear stress applied to Caco-2 cells, peristalsis-like motions is guaranteed by the attachment of the membrane to two hollow chambers that rhythmically are inflated and deflated with air. These motions induce the differentiation of Caco-2 cells with the formation of intestinal villi by epithelial cells of the small intestine (i.e., absorptive, goblet, enteroendocrine, and Paneth; Kim et al., 2012). This device is, at the current state of the art, one the most advanced *in vitro* models of the gut epithelium-microbiota interface, since it has all the necessary features. Among these last ones, there are the dynamic environment for both cells and bacteria, capability to sustain the oxygen gradient for strict anaerobic bacteria, presence of peristalsis, optical accessibility, presence of oxygen sensors and TEER electrodes for non-invasive measurements, potentiality to host primary cells together with harvested human complete microbiota. Moreover, this kind of system could be also hydraulically connectable with other organ-on-a-chip devices, making it suitable for also body-on-a-chip purposes.

#### Human-Microbial CrossTalk (HuMiX) System

The HuMiX is the most recently described aerobic-anaerobic co-culture system. It is a microfluidic device composed of a modular stacked assembly of elastomeric gaskets, sandwiched between two polycarbonate enclosures, where each gasket defines a spiral-shaped micro-channel. The upper compartment (microbial micro-chamber) is separated from the middle compartment (epithelial cell micro-chamber) by a nanoporous membrane, while the bottom microchannel (perfusion micro-chamber) is separated from the epithelial cell micro-chamber by a microporous membrane. The viability of the co-culture is measured by an integrated TEER measurement system and the oxygen concentration is monitored through non-invasive sensors located in each compartment. In the compartments there are inlets and outlets areas that allow the arrival of substances and the removal of sample for downstream analyses. Among the compartments there are semi-permeable polycarbonate membranes that have different pore sizes. The membrane with larger pores is placed between the microbial and epithelial compartment, while the membrane with smaller pores is placed between the perfusion and the epithelial area. The membranes are coated with mucin and collagen during co-culture to promote cell adhesion. During incubation, standard cell culture medium is perfused through the compartments to facilitate dynamic cell growth and thus better resemble the *in vivo* situation (Shah et al., 2016). Co-culture experiments with the HuMiX system have been conducted (Eain et al., 2017). Two variants of the system have been developed: (a) Immuno-HuMiX, and (b) Nutri-HuMiX. Immuno-HuMiX integrate the immune-component (CD4 T cells, Th1 and Th2 cells) to study the interaction between the immune system and gut microbiota, while Nutri-HuMiX is used to study the effect of diet on one or more bacterial components and on intestinal epithelial cells (Eain et al., 2017). This system has great potential, it can host viable cells for 7 days (for the last 24 h in co-culture with the commensal *Lactobacillus rhamnosus GG*). Although the presence of a dynamic environment, with respect to the previously described gut-on-a-chip device developed by Kim et al. (2012), it lacks the possibility to integrate a system which provides peristaltic movements to the intestinal cells. The structure of the chamber and its amplitude have been chosen to have a correct chamber priming, enough surface material for cell growth and analysis at the microscope. The presence of well-integrated sensors and electrodes, together with the optical accessibility of the device, make the system non-invasive. The model could be enhanced to host the complete human microbiota.

#### The Microfluidic Cell Culture Device (μFCCD)

μFCCD is composed of five PDMS layers with different thickness and a polyester porous membrane. The inlet of the flow channel has bubble trap chambers to prevent holes from being blocked with bubbles. Nutrients to the cells in the channel are provided and laminar flow allows uptake and transport of nutrients. Caco-2 cells were cultured on a porous membrane for 3 days and under a low flow rates (0.5 μl/min) an increase in production of mucin was noticed. MUC-2, the major mucin produced in the small and large intestine, covers the apical surface of intestinal tissue. Caco-2 cells cultured in this microfluidic system differentiate in villi-like and crypt-like structures and have high aminopeptidase activity, which breaks down small peptides to amino acids allowing the reabsorption of amino acids by intestinal cells. The intestinal cells grown in the μFCCD are shown to be tightly connected to each other, metabolically active, and have a large surface area for absorption. A simulation of bacterial invasion in the model was performed inoculating in the μFCCD the *Salmonella enterica serovar Typhimurium (*χ*3339)* (Chi et al., 2015). The device was one of the first to be able to offer a double dynamic perfusion to the cells cultured on a porous membrane, in fact, the medium flowed on the lower and upper chamber of the device. Moreover, its optical accessibility and compatibility with TEER measurements made the device non-invasive. An implementation of this device with the possibility of hosting the complete human microbiota together with primary intestinal cells may be an interesting technical improvement.

#### Shim's Microfluidic Gut-on-a-Chip

The microfluidic gut-on-a-chip described by Shim et al. (2017) consists of three layers of PDMS, a slide glass and a polyester membrane which was manually separated from Transwell clear inserts. The first layer provides reservoirs for cell culture media, the second layer has fluidic channels connecting to the apical side of the gut with a hole at the centre and four holes at the corners, while the third layer has an hole at the centre and two holes at the corners. The 3D villi were obtained by photolithography and bonded to a porous membrane, fixed inside the PDMS chip, using collagen. Caco-2 cells were cultured in the chip for 14 days to induce differentiation. The microfluidic environment shortened the time required for complete differentiation (3 to 5 days) and induced the formation of tight junctions. Moreover, 3D villi scaffolds improved Caco-2 cell metabolic activity (Shim et al., 2017). The device offers a dynamic, optically accessible environment, capable to induce cell growth on the 3D villi scaffolds and compatible with TEER measurements. However, the model has several limitations, including the use of Caco-2 immortalized cells (that do not produce mucus and do not represent *in vivo* conditions). Again, this gut-on-a-chip can be a basis for improvements by adding the microbiota or its secreted molecules, thus realizing an *in vitro* advanced device to study microbiota-gut communication.

#### Multi-Organ-on-a-Chip

The last and most advanced organ-on-a-chip designed to study microbe-human cells interactions features bacterial and different human cells in an attempt of increasing complexity of the above described solutions. It is composed of an optically clear chip made by flexible PDMS polymer, divided into two chambers (aerobic and anaerobic) with inlets, middles and outlets of the top and bottom channels, embedding six oxygen-quenched fluorescent particles to monitor in real time the levels of oxygen in the system. The anaerobic chamber is continuously flushed with CO_2_-N_2_ gas mixture and can be maintained low oxygen levels (<5 %) for 15', while in the bottom vascular channel an oxygenated medium supplies oxygen to the endothelium that covers its surface. Caco-2 cells are cultured for approx. 5–7 days under dynamic flow, allowing cells to differentiate and express features of the ileal portion of the human small intestine, including villus construct, mucus secretion, and cytokine release. Intestine Chips lined with Caco-2 cells and human intestinal microvascular endothelial cells (HIMECs) were tested under a hypoxia gradient, showing the ability to differentiate and remain viable for about 7 days. The system was validated using co-culture of the intestinal epithelium with the obligate anaerobe *Bacteroides fragilis NCTC 9343* under hypoxic environment. The epithelium has been previously cultured and differentiated on-chip for 7 days under anaerobic conditions and then bacteria were introduced into the lumen of the upper channel on the surface of the intestinal epithelium, under either aerobic or anaerobic conditions. After 3 days the environment showed a low level of oxygen, despite this Caco-2 cells continued to grow maintaining the functionality of the tight junctions and apical brush border polarity. The Caco-2 barrier function was maintained for at least 8 days in culture, while the *B. fragilis* bacteria continued to grow in anaerobic chips for about 3 days under anaerobic conditions and for about 2 days under aerobic conditions. The system was also tested using cells from surgical biopsies of human ileum (i.e., organoid) that have maintained functions and abilities also when grown on-chip and a complex gut microbiome from fresh human feces. The chip stably maintained for about 5 days the epithelial barrier function and the bacterial richness (Jalili-Firoozinezhad et al., 2019). This system is an advanced tool prone to many applications in the field of microbiota-gut crosstalk and may be further implemented as part of a multi-organ-on-a-chip platform. At the current state of the art, it is the most forefront organ-on-a-chip device able to model the interface between the aerobic human epithelial cells and the anaerobic human gut-microbiota. From a technological point of view, it does not lack any of the main features that are required for an intestine-on-a-chip model to be defined as complete. Among these features, there are the presence of a dynamic environment for both cells and bacteria, capability to sustain the oxygen gradient for strict anaerobic bacteria, presence of peristalsis, optical accessibility, presence of oxygen sensors and TEER electrodes for non-invasive measurements, capability to host mucus producing and villi-forming primary cells, together with harvested human complete microbiota.

## Conclusions

During the last 10 years, the medical and scientific community have carried out a great re-examination of the role of resident microorganisms in our body in human patho-physiology, with particular attention to those living in the intestine. We have several evidence confirming that aging, diet, environmental factors, and lifestyle can affect the composition of our intestinal microbiota, with implications not only for its habitat, but also on a systemic level and up to the brain. The latter aspect, postulated by the “microbiota-gut-brain axis” hypothesis, correlates abnormalities of this huge and diversified microbial community with the occurrence also of neurodegenerative processes, including AD and PD. Despite the animal models remain one of the most widespread choice for the study of these complex multi-organ interactions, we do need scientific advances and clinical impact.

As detailed in the second part of this review, the use of bioengineering and material science may have a role to provide tools useful to detect the possible causative role of gut microbiota on brain diseases, by fabricating reliable and reproducible models to host microbial and human gut cells at the same time as an essential step to collect information about the microbiota and how its works in producing metabolites that may trigger neurodegeneration. To better mimic body environment, solutions such as 3D cell architecture simulated by appropriate physiological cues, cell–cell interfaces that restore native tissue architecture and function and the recapitulation of specific morphological, mechanical and biochemical organ properties are important improvements to be achieved. To this respect, rapid advances in *in vitro* technologies, especially organ-on-a-chip models, are promising fields for potential replacement (or at least reduction) of *in vivo* experiments. In fact, the increasing complexity of organ-on-a-chip models, that include channels mimicking microvascular endothelium and physiological flows, cyclic mechanical forces that recall the peristalsis-like deformations of the intestine, along with new strategies to keep anaerobic microorganisms alive, pushed the study forward aspects related to host-microbes interactions, even though we are still far from a model that is able to simulate the full relationship among gut-microbiota-brain.

To achieve an organ-on-a-chip-based model that recapitulates all aspects of the microbiota-gut-brain axis, the next advanced systems should follow a multi-organ perspective and incorporate intestinal epithelial, endothelial, stromal, neuronal, and immune cell types, in addition to the microbiota component, in order to feature all the players of the axis. We should remember one other important player of the microbiota-gut-brain axis: the liver, which performs many essential functions related to digestion and metabolism, as processing, storage, alteration, and detoxification of bioactive molecules, including several neuroactive compounds produced in the gut (Mancini et al., 2018; Boeri et al., 2019). In fact, many bacterial-derived metabolites are indeed processed by the liver and pass back into the blood or are released into the intestine to be eliminated.

This multi-organ-on-a-chip approach is peculiar of the MINERVA project, based on the serial connection of organ-on-a-chip devices. Each device is a miniaturized, microfluidic, optically accessible organ-on-a-chip recapitulating key features of the microbiota, the epithelial barrier, the immune system, the BBB, and the brain.

Each of the above-mentioned compartments will permit both cell perfusion and sampling before and after the culture chamber. A microporous membrane will divide each chamber into two parts, separating the cells from culture medium flowing to the next device. Therefore, the culture medium will be initially enriched with the secretome produced by the gut microbiota and then after flowing through the gut epithelial cells, immune cells and BBB cells, it will reach the brain cells. Therefore, the microbiota compartment will be represented by the first device of the platform and will be designed to have a hydrogel simulating the intestinal mucus encapsulating the gut microbiota, harvested from fecal samples from healthy or pathological donors. A parallel flow, running on the opposite side of the membrane will collect the secretome, which must flow through the platform. In particular, gut epithelial cells plated on a microporous membrane, lymphocytes and macrophages in suspension, EC and astrocytes plated on both sides of a microporous membrane and a 3D hydrogel with embedded neurons, astrocytes and microglia will respectively model the gut barrier, the immune system, the BBB and the brain. A 3D hydrogel modeling brain ECM will embed neurons, astrocytes and microglia (as single cell cultures or as a co-culture). The MINERVA design and concept has been thought as a technological improvement of a miniaturized, optically accessible bioreactor developed for the interstitial perfusion of 3D cell constructs (Izzo et al., 2019). The resulting MINERVA platform may be an innovative tool for clinicians and researches and a contribution in the field of microbiota's role in brain neurodegenerative disorders (Raimondi et al., 2019).

## Author Contributions

FC, DA, and CG were involved with the conception and design of the manuscript. LI, LS, IR, and MT contributed parts of writing. All authors contributed to the article, revised the manuscript, and approved the submitted version.

## Conflict of Interest

The authors declare that the research was conducted in the absence of any commercial or financial relationships that could be construed as a potential conflict of interest.

## Abbreviations

5-MTHF: 5-methyltetrahydrofolate
ACTH: AdrenoCorticoTropic Hormone
AD: Alzheimer's Disease
APP: Amyloid Precursor Protein
Aβ: Amyloid beta
ALS: Amyotrophic Lateral Sclerosis
AMPs: Anti-microbial Peptides
ANS: Autonomic Nervous System
BBB: Blood-Brain Barrier
CNS: Central Nervous System
CCK: CholeCystoKinin
CRF: Corticotropin-Releasing Factor
SIMGI: Dynamic GastroIntestinal Simulator
ENS: Enteric Nervous System
ECM: Extracellular Matrix
FOS: Fructooligosaccharides
GABA: γ-AminoButyric Acid
GI: gastrointestinal
GF: germ-free
GLP-1: Glucagon-Like Peptide 1
GALT: Gut-Associated Lymphoid Tissue
HMI: Host-Microbiota Interaction module
SHIME: Human Intestinal Microbial Ecosystem
HIMECs: human intestinal microvascular endothelial cells
HMA: human microbiota-associated
HuMiX: Human-Microbial cross talk
HPA: Hypothalamic-Pituitary-Adrenal
IL: Interleukin
IECs: Intestinal Epithelial Cells
IBS: Irritable Bowel Syndrome
LPS: LipoPolySaccharides
MAMPs: Microbial-Associated Molecular Patterns
μFCCD: Microfluidic cell culture device
NPY: NeuroPeptide Y
NO: nitric oxide
PNS: Parasympathetic Nervous System
PD: Parkinson's Disease
PRRs: Pattern Recognition Receptors
PYY: Peptide YY
PDMS: polydimethylsiloxane
RNS: Reactive Nitrogen Species
ROS: Reactive Oxygen Species, 5-HT, Serotonin
SCFA: Short-Chain Fatty Acid
SPF: specific pathogen-free
SNpc: Substantia Nigra pars compacta
SNS: Sympathetic Nervous System
TIM: TNO Gastro-Intestinal Model
TLR: Toll-like receptor
TRP: Tryptophan, TPH1, Tryptophan hydroxylase 1
TNFα: Tumor Necrosis Factor-α
IFNs: type I interferons
YLDs: Years Lived with Disability
α-syn: α-synuclein
BMAA: β-N-methylamino-L-alanine
RgpA: Arginine Gingipain A
RgpB: Arginine Gingipain B
CSF: Cerebrospinal Fluid
EEC: Enteroendocrine cells
Kgp: Lysine-Gingipain
SIEM: Simulated Ileal Efflux Medium
TDC: Tyrosine Decarboxylase
TEER: transepithelial electrical resistance.
